# Effects of Goji Berry Supplementation on Immune-Related and Antioxidant Gene Expression in the Male Rabbit Reproductive Tract

**DOI:** 10.3390/ani15131921

**Published:** 2025-06-29

**Authors:** Alda Quattrone, Susanna Draghi, Alessia Inglesi, Federica Riva, Luigj Turmalaj, Joel Filipe, Majlind Sulçe, Stella Agradi, Daniele Vigo, Gerald Muça, Laura Menchetti, Enkeleda Ozuni, Olimpia Barbato, Nour Elhouda Fehri, Marta Castrica, Gabriele Brecchia, Giulio Curone

**Affiliations:** 1 Department of Veterinary Medicine and Animal Sciences, University of Milan, Via dell’Università 6, 26900 Lodi, Italy; alda.quattrone@unimi.it (A.Q.); susanna.draghi@unimi.it (S.D.); alessia.inglesi@unimi.it (A.I.); joel.soares@unimi.it (J.F.); daniele.vigo@unimi.it (D.V.); nour.fehri@unimi.it (N.E.F.); giulio.curone@unimi.it (G.C.); 2 Faculty of Veterinary Medicine, Agricultural University of Tirana, Kodër Kamëz, 1029 Tirana, Albania; l.turmalaj@ubt.edu.al (L.T.); msulce@ubt.edu.al (M.S.); gmuca@ubt.edu.al (G.M.); enkeleda.ozuni@ubt.edu.al (E.O.); 3 Department of Veterinary Sciences, University of Torino, Largo Paolo Braccini 2, 10095 Grugliasco, Italy; stella.agradi@unito.it; 4 School of Biosciences and Veterinary Medicine, University of Camerino, Via Circonvallazione 93/95, 62024 Matelica, Italy; laura.menchetti@unicam.it; 5 Department of Veterinary Medicine, University of Perugia, Via San Costanzo 4, 06126 Perugia, Italy; olimpia.barbato@unipg.it; 6 Department of Comparative Biomedicine and Food Science, University of Padova, Agripolis, Viale dell’Università 16, 35020 Legnaro, Italy; marta.castrica@unipd.it

**Keywords:** Goji berry, male reproductive tract, epididymis, cytokines, oxidative status, gene expression, rabbit buck, Toll-like receptor, TLR4, GPX

## Abstract

Goji berries (*Lycium barbarum*), rich in antioxidant and immunomodulatory compounds, have shown potential benefits for male reproductive health. This study aimed to evaluate the impact of dietary Goji berry (GB) supplementation on immune-related and antioxidant gene expression in the male reproductive tract of rabbits. Eighteen 7-month-old New Zealand White rabbit bucks were randomly assigned to two groups: a control group (*n* = 9) receiving a standard diet, and a Goji group (*n* = 9) receiving the same diet supplemented with 1% GB. After 60 days of nutritional adaptation and then 60 days of treatment, tissues from the testes, epididymis, seminal vesicles, prostate, and bulbourethral glands were collected and analyzed using quantitative real-time PCR. Gene expression analysis focused on immune markers (TLR4, IL-1β, IL-10, and TNFα) and antioxidant enzymes (SOD1, CAT, and GPX). Significant modulation was observed only in the epididymis, where TLR4 and GPX were significantly downregulated in the Goji group (*p* = 0.0274 and *p* = 0.007, respectively), while IL-1β and TNFα showed a downward trend. No significant differences were found in the other tissues. These results suggest that Goji berry supplementation exerts tissue-specific effects, particularly in the epididymis, by modulating inflammation and oxidative stress. This supports its potential use as a natural nutraceutical strategy to enhance male fertility in rabbits.

## Simple Summary

Male fertility is influenced by various factors, including inflammation and oxidative stress, which can negatively affect sperm quality. Goji berries, known for their natural antioxidant and immune-supporting properties, may offer benefits for reproductive health. In this study, we investigated whether including Goji berries in the diet of healthy male rabbits could improve the immune and antioxidant status of their reproductive organs. Different parts of the male reproductive system were examined to assess changes in the expression of genes involved in inflammation and oxidative balance. Our results showed that, in the epididymis, the key organ where sperm matures and is stored, the addition of Goji berries to the diet led to a reduced expression of genes related to inflammation and oxidative stress. These effects were not observed in other reproductive organs, such as the testes or prostate. The findings suggest that Goji berries may help create a more favorable environment for sperm development, potentially supporting male fertility. This study highlights the potential of Goji berries as a natural dietary supplement to protect reproductive health in animals, with possible future applications in improving fertility in both veterinary and human medicine.

## 1. Introduction

Superfruits are rich in phytochemical compounds and offer numerous health benefits [1]. Among them, Goji berries (GBs), also known as wolfberries, are the fruits of *Lycium barbarum* and *Lycium chinense*, two closely related species in the Solanaceae family [2]. Native to China, they have long been used in traditional Chinese medicine as natural nutraceuticals [3,4]. In recent years, GBs have gained popularity in North America and Europe due to their reported health-promoting effects, including anti-aging [5]; anti-inflammatory [6]; anti-cancer [7,8]; and potential benefits in glaucoma management [9], liver protection, immune support [10,11], gastrointestinal protection [12], and male fertility enhancement [13,14,15]. GBs are a rich source of vitamin C [16]; essential minerals such as iron, zinc, selenium, and copper; antioxidants [17]; amino acids [18]; and dietary fiber [19]. These components help neutralize free radicals and support energy homeostasis [20], essential for reproductive and overall health. Among their bioactive compounds, the most studied are *Lycium barbarum* polysaccharides (LBPs), which are water-soluble, highly branched molecules constituting 5–8% of the dried fruit’s weight [21]. LBPs are primarily responsible for GBs’ biological activities, including antioxidant, anti-aging, immunoregulatory, antitumor, neuroprotective, and antidiabetic effects [22,23,24], making them central to the berries’ therapeutic potential [21]. Thanks to their antioxidant and immunomodulatory properties, GBs have attracted interest for their potential role in enhancing male fertility [25]. Supplementation with GBs may improve sperm quality and reproductive health by reducing oxidative stress, a major factor impairing sperm function [25]. Several studies report improvements in semen parameters such as sperm concentration, motility, and hormone levels across species [21]. In laboratory animals, particularly rats, LBPs have been shown to enhance sexual performance [7,26], improve sperm quality [27], and protect testicular tissue from damage by preserving seminiferous epithelium integrity and preventing apoptosis [14,26]. LBP supplementation has been found to restore testosterone levels in hemicastrated or irradiated rats, highlighting its potential role in endocrine regulation [27,28]. Notably, LBPs also contribute to the modulation of sexual behavior by promoting neurogenesis, which may be a key mechanism underlying their positive effects on sexual function [29].

Beyond reproductive health, LBPs have demonstrated immunomodulatory effects in various species, including humans [15], broilers [30], piglets [31], sheep [32], and rodents [11,33]. These effects are mediated through cytokine secretion and immune cell activation [11,33]. In a recent study, intragastric administration of LBPs in mice with impaired reproductive systems increased blood levels of TNF-α, suggesting a potential immunomodulatory activity and improved overall health by stimulating the expression of pro-inflammatory mediators, which protect epididymis and sperm [33].

In rabbits, dietary GB supplementation has been associated with significant improvements in semen quality parameters [34]. Specifically, rabbit bucks fed 1% GB supplementation exhibited notable enhancements in sperm concentration, motility, and vitality compared to the control group. Additionally, there was a significant reduction in the percentage of abnormal spermatozoa and an observed increase in libido among the GB-supplemented rabbits. These positive changes were associated with improved histological features of the reproductive tract, suggesting that GB may exert beneficial effects on reproductive tissues. Building on these promising findings, we selected the same 1% dietary inclusion level of GB for our study. This dosage was based on previous research conducted in both male and female rabbits that demonstrated beneficial effects on various reproductive and productive parameters [34]. Notably, this supplementation level was well tolerated by male rabbits, with no health issues observed, and the feed was readily consumed, indicating good palatability [34]. Moreover, the 1% inclusion rate is cost-effective and practical for application in breeding programs, supporting its potential as a natural strategy to enhance reproductive performance in rabbits.

Despite these promising results, research on GBs’ effects on male rabbit reproductive health remains limited. While numerous studies have investigated dietary strategies to support female rabbit reproduction [20,35], less attention has been given to males. Importantly, no studies to date have explored how GB supplementation influences oxidative stress, immune response, and inflammation at the molecular level in the male rabbit reproductive system. Given the rabbit’s high reproductive efficiency and its value as an experimental model [36], understanding the molecular effects of GBs on gene expression related to immunity, oxidative stress, and inflammation could provide valuable insights into male fertility in both veterinary and human reproductive research.

This study therefore aims to evaluate the impact of GB supplementation on the expression of key genes involved in immune regulation, oxidative balance, and inflammation within the male reproductive tract of rabbit bucks, addressing a significant gap in current research.

## 2. Materials and Methods

### 2.1. Animals, Diets, and Sample Collection

The study was conducted at the Faculty of Veterinary Medicine, Agricultural University of Tirana, Albania, following approval from the Albanian Ministry of Agriculture and Rural Development, National Authority of Veterinary and Plant Protection (protocol number 824/2021). Measures were implemented to minimize animal discomfort, and the rabbits’ health and welfare were monitored daily. Eighteen 7-month-old New Zealand White rabbit bucks were individually housed under controlled environmental conditions and a standardized lighting regimen. After a 60-day nutritional adaptation period, the rabbits were randomly assigned to two dietary groups: control (*n* = 9), receiving a standard pelleted diet; and Goji (*n* = 9), receiving the same diet supplemented with 1% Goji berries. This nutritional adaptation phase ensured that all animals adjusted to the diets and reached a stable physiological state, minimizing variability due to prior dietary influences. During the experimental trial, which lasted another 60 days, each rabbit was provided 150 g/day of feed, with water available ad libitum. The diet compositions are detailed in Table 1 and Table 2 and are consistent with formulations used in previous research [34].

At the end of the experimental trial, rabbits were sacrificed, and their reproductive tissues were collected. Specifically, 0.5 cm^3^ samples of the testes, epididymis, seminal vesicles, prostate, and bulbourethral glands were excised and placed in 2 mL sterile tubes containing RNAlater^®^ (Sigma-Aldrich, St. Louis, MO, USA) to preserve RNA integrity. Testis samples were obtained by excising a full-thickness cylindrical section representative of the entire organ through a longitudinal incision along its short axis, ensuring inclusion of all anatomical layers, whereas the epididymis was sectioned at the cauda (tail) region. The samples were then stored at −20 °C until analysis.

### 2.2. RNA Extraction from Rabbit Reproductive Tissues, Reverse Transcription, and Real-Time PCR

RNA extraction from the testes, epididymis, seminal vesicles, prostate, and bulbourethral glands was performed using TRI Reagent (Sigma-Aldrich, St. Louis, MO, USA), following the manufacturer’s instructions. The concentration and purity of the extracted RNA were assessed using a spectrophotometer (BioPhotometer, Eppendorf, Hamburg, Germany) at a wavelength of 260 nm. From each sample, 2 µg of total RNA was reverse-transcribed into complementary DNA (cDNA) using the High-Capacity cDNA Reverse Transcription Kit (Applied Biosystems, Foster City, CA, USA). Real-time PCR was then conducted in a 25 µL optimized reaction volume using SYBR Green (Applied Biosystems, Foster City, CA, USA). The specific primer pairs for rabbit TLR4, IL-1β, IL-10, TNFα, SOD1, CAT, and GPX were designed using Pimer3Plus^®^ (version: 3.3.0) and synthesized by Eurofins (Luxembourg City, Luxembourg). Rabbit β-actin (ACTB) was used as the housekeeping gene. The primer sequences are listed in Table 3. For each sample, reactions were performed in duplicate, and no-template controls were included for each primer pair in every plate to ensure specificity and detect potential contamination. Real-time PCR was performed in Applied Biosystems™ QuantStudio™ 5 Real-Time PCR System (Applied Biosystem, Foster City, CA, USA). The calculated ACTB cDNA expression of the same sample and run was used to normalize the rabbit target genes. The method of “delta Ct” described by Schmittgen and Livak [38] was used to calculate the relative quantification; and finally, the obtained values were multiplied by 10,000 to obtain the arbitrary units (AU).

### 2.3. Statistical Analysis

Statistical analyses were performed using GraphPad Prism 8 (La Jolla, CA, USA). At first, Shapiro–Wilk tests and diagnostic graphics were used to verify the distribution of data and the equality of variances. The differences in gene expression between the 2 groups (control diet and supplemented with Goji) were analyzed using Mann–Whitney tests. The values of *p* were considered statistically significant when *p* < 0.05 and tendencies at *p* ≤ 0.10.

## 3. Results

### 3.1. Goji Berry Supplementation Modulates Immune-Related Gene Expression in the Epididymis

**Figure 1:**
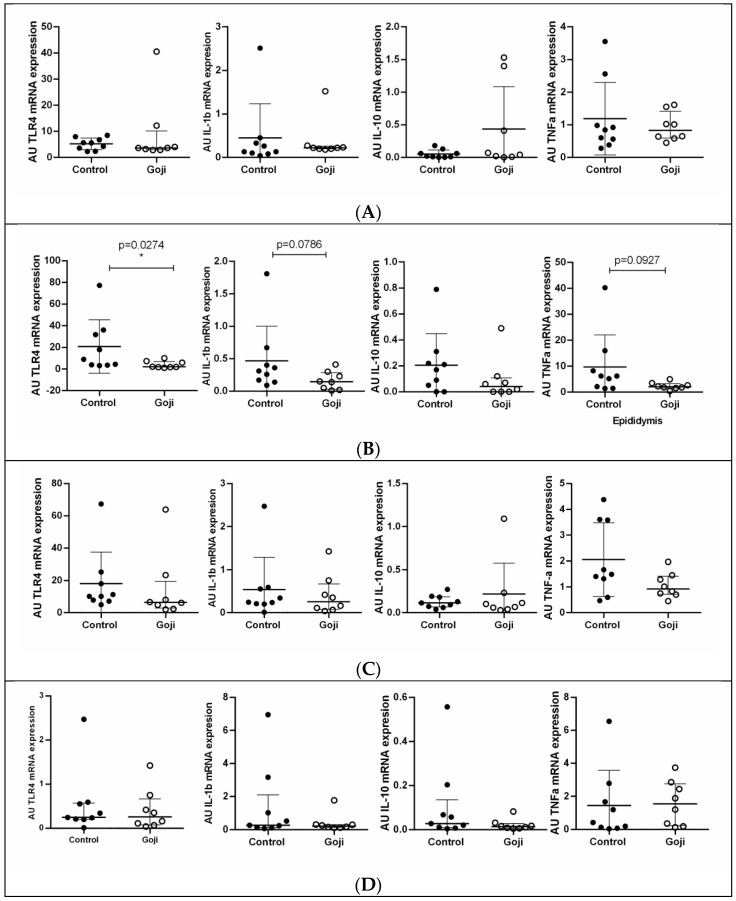

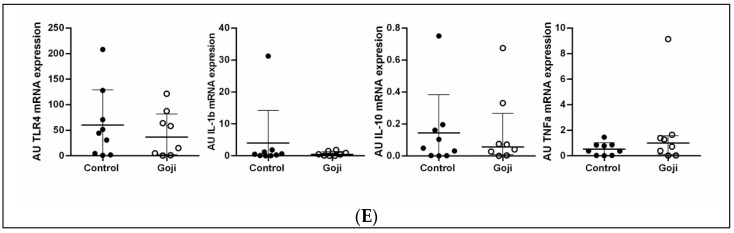
Gene expression of immune-related molecules (TLR4, IL-1β, IL-10, TNFα) in the reproductive tract of male rabbits: (**A**) testes; (**B**) epididymis; (**C**) seminal vesicles; (**D**) bulbourethral glands; and (**E**) prostate. Results are presented as arbitrary units (AU). The charts show individual values; horizontal lines indicate medians and interquartile ranges in all panels, with * *p* < 0.05.

As illustrated in Figure 1A,C,D, no statistically significant differences were observed in the expression of immune-related genes between the control and GB-supplemented groups in the testes, seminal vesicles, bulbourethral glands, and prostate. However, in the epididymis (Figure 1B), TLR4 expression was significantly downregulated in the Goji group compared to the control group (*p* = 0.0274). Additionally, IL-1β and TNFα exhibited a trend toward lower expression in the Goji group, although the differences did not reach statistical significance (*p* = 0.0786 and *p* = 0.0927, respectively; Figure 1B). No significant differences were detected in IL-10 expression between the two groups in any of the investigated reproductive-tract tissues.

### 3.2. Goji Berry Supplementation Modulates Antioxidant-Related Gene Expression Only in the Epididymis

**Figure 2:**
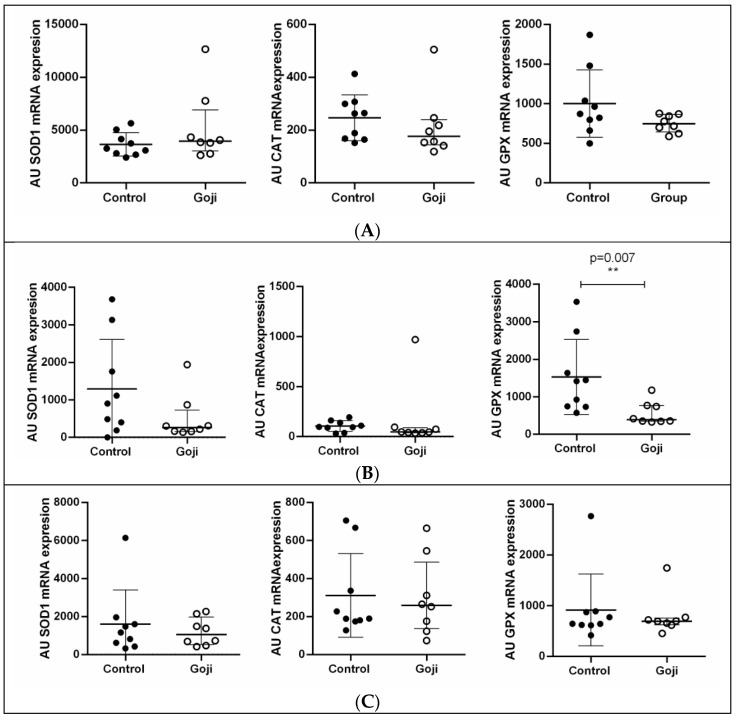

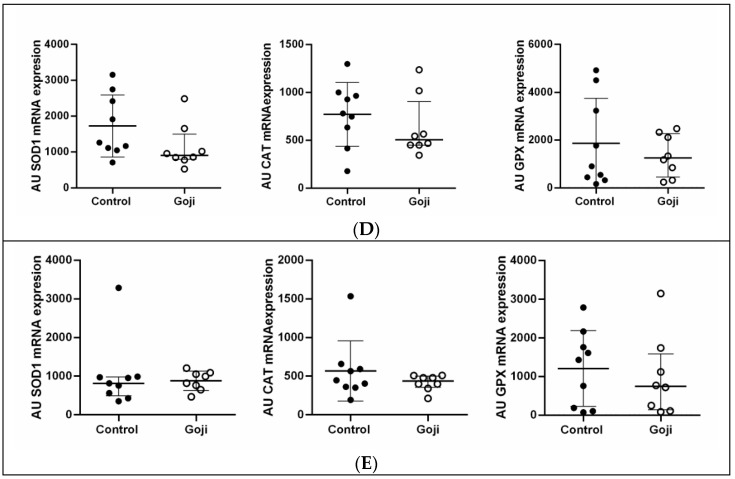
Gene expression of antioxidant-related molecules (SOD, CAT, and GPX) in the reproductive tract of male rabbits: (**A**) testes, (**B**) epididymis, (**C**) seminal vesicles, (**D**) bulbourethral glands, and (**E**) prostate. Results are presented as arbitrary units (AU). The charts show individual values; horizontal lines indicate medians and interquartile ranges in all panels, with ** *p* < 0.01.

As shown in Figure 2A,C–E, dietary Goji berry supplementation had no significant effect on the expression of SOD1, CAT, and GPX in the testes, seminal vesicles, bulbourethral glands, or prostate. However, in the epididymis (Figure 2B), GPX expression was significantly lower in the Goji group compared to the control group (*p* = 0.007).

## 4. Discussion

This research explored, for the first time, the effects of Goji berry supplementation on the immune-related gene expression and antioxidant activity of the male reproductive system in rabbit bucks. Goji berries, recognized for their potential antioxidant and immunomodulatory properties, have been previously shown to affect reproductive health across various species, including rabbits [39,40,41,42,43]. Our findings revealed that the expression of immune-related genes and antioxidant enzymes was predominantly modulated in the epididymis, while no significant changes were observed in other tissues, such as the testes, seminal vesicles, bulbourethral glands, and prostate. The epididymis is essential for both sperm maturation and storage, and it plays a critical role in defending against pathogens ascending from the lower reproductive tract [44]. This dual function necessitates a finely balanced immune environment to safeguard against infections while maintaining sperm integrity, which could otherwise provoke immune responses due to its antigenic nature [45]. Epididymal epithelial cells (EECs) are integral to this balance, expressing various pattern recognition receptors (PRRs), including Toll-like receptors (TLRs), to identify and respond to pathogens [46]. Notably, TLR4 (Toll-like receptor 4) is one of the PRRs expressed by EECs, enabling the binding of lipopolysaccharides (LPSs) from Gram-negative bacteria and initiating innate immune responses [45,47]. TLR4 is a key mediator of innate immunity, especially in detecting microbial pathogens and activating inflammatory pathways through the activation of nuclear factor kappa B (NF-κB) [48,49], which subsequently stimulates the expression of proinflammatory cytokines such as IL-1β, TNF-α, and IL-6 [50]. Additionally, TLR4 activation promotes the production of inflammation-related mediators, including reactive oxygen species (ROS); nitric oxide; and antimicrobial factors like defensins and heat shock proteins [51,52,53]. In both humans and animals, inflammatory conditions in the reproductive tract, such as epididymitis, can significantly impair sperm maturation and function, leading to decreased motility, abnormal morphology, and reduced sperm viability, thus negatively impacting fertilization potential [54,55]. Chronic inflammation may also result in the scarring of the epididymal ducts, obstructing sperm flow and causing conditions such as oligospermia or azoospermia, ultimately reducing fertility [56,57]. In particular, rabbit genital infections are commonly caused by Gram-negative bacteria such as *Pasteurella multocida*, *Escherichia coli*, *Pseudomonas* spp., and *Salmonella* spp., often associated with improper artificial insemination practices and poor environmental conditions [47,58]. TLR4, which plays a crucial role in the innate immune response by recognizing and binding to LPS from these ascending Gram-negative bacteria, in rabbits, has been detected in various parts of the reproductive tract [58], with notably higher expression levels observed in the epididymis and seminal vesicles; meanwhile, its presence in the testes and prostate appears more variable [47]. This pattern suggests a tissue-specific function for TLR4 in immune surveillance at the reproductive level, helping protect against infections that could compromise fertility [58]. Comparatively, in humans, TLR4 expression is also observed throughout the male genital tract, including the epididymis and prostate [59]. The functional similarities between rabbit and human TLR4, alongside their genetic similarities, highlight the potential of rabbits as a valuable model for studying TLR4-mediated immune responses in the male reproductive system [59]. This resemblance enhances the translational relevance of rabbit-based research, providing insights that may be applicable to understanding human reproductive immunology and associated pathologies. Despite differences in the spatial distribution of TLR4 across species, its widespread expression suggests that the male reproductive tract is constantly under immune surveillance, with TLR4 playing a crucial role in responding to infections [60,61].

In our study, the most notable finding was the downregulation of TLR4 in the epididymis of rabbits fed a Goji-supplemented diet. The observed downregulation of TLR4 could be attributed to the effect of *Lycium barbarum* polysaccharides (LBPs), which, as demonstrated by several studies, can modulate the TLR4/NF-κB signaling pathway [62,63]. LPBs prevent the degradation of IκBα, a key inhibitor of NF-κB, thus blocking the downstream inflammatory cascade [64]. Additionally, IL-1β and TNFα, both important pro-inflammatory cytokines, tended to be less expressed in the Goji group, though not reaching statistical significance. This trend is consistent with the anti-inflammatory properties attributed to Goji berries and suggests that the supplementation may help reduce inflammatory processes in the male reproductive system [34,41]. The observed trends toward reduced IL-1β and TNFα expression further support the notion of GB’s anti-inflammatory properties, as these cytokines are key mediators of inflammation [65]. Interestingly, the absence of significant changes in IL-10 expression, an anti-inflammatory cytokine, suggests that GBs’ modulatory effects might have a more pronounced effect on downregulating pro-inflammatory pathways.

Goji berries are also widely recognized for their powerful antioxidant properties [66], which stem from the synergistic effects of various bioactive compounds, including polysaccharides, flavonoids, phenolic acids, and carotenoids [67]. These compounds function through multiple mechanisms to neutralize free radicals and reactive oxygen species (ROS), thereby protecting cellular structures from oxidative damage and reducing the risk of pathological conditions [68]. In vitro studies have confirmed that GB extracts efficiently scavenge different types of free radicals [69]. Notably, certain compounds derived from GB exhibit a strong ability to neutralize hydroxyl and superoxide radicals, showing antioxidant activity comparable to that of well-established compounds. Specifically, their superoxide radical scavenging ability parallels that of superoxide dismutase [70]. Additionally, animal studies have reinforced the in vivo antioxidant effects of GB. For example, rats supplemented with GB extracts displayed increased activity of key antioxidant enzymes, including superoxide dismutase (SOD), catalase (CAT), and glutathione peroxidase (GPX), at the systemic level [71].

Our study revealed a significant reduction in the expression of GPX (glutathione peroxidase) in the epididymis, a key enzyme involved in protecting cells from oxidative stress [72]. This finding is particularly interesting, given that, as already mentioned, the epididymis is the site for sperm maturation, transport, and storage [73]. At this level, reactive oxygen species (ROS) are natural byproducts of cellular redox metabolism, and at moderate levels, they contribute to sperm maturation and capacitation [73]. However, sperm cells are highly vulnerable to oxidative damage due to their membranes being rich in polyunsaturated fatty acids (PUFAs), which are particularly prone to peroxidation [74]. This sensitivity is even more pronounced in rabbits, as their sperm membranes naturally contain elevated levels of long-chain PUFAs [75,76]. Therefore, maintaining optimal ROS levels is critical for ensuring normal sperm function. Since spermatozoa lose most of their cytoplasm during maturation, their intrinsic antioxidant capacity is limited [77]. To counteract oxidative stress, epididymal epithelial cells produce antioxidant enzymes such as catalase, superoxide dismutase, and glutathione peroxidases (GPXs) [77,78]. GPXs play a particularly significant role, as they are not only present in the sperm plasma membrane but also closely associated with spermatozoa throughout their journey in the excurrent duct system [73]. Among them, GPX5, primarily synthesized by the caput epididymis, is secreted into the luminal fluid to protect immature sperm from oxidative damage [73]. In addition to neutralizing hydrogen peroxide (H_2_O_2_), GPXs eliminate various oxidative substrates, including organic peroxides and peroxynitrite anions [73,79]. By preserving the epididymal microenvironment and shielding spermatozoa from oxidative damage, GPX enzymes contribute significantly to maintaining sperm DNA integrity and overall functionality [80].

The lower expression of GPX in the Goji-supplemented group suggests that the antioxidant environment in the epididymis may have improved, reducing the need for high levels of endogenous antioxidants [81]. This reduction in GPX could reflect a decreased oxidative burden in the cells, likely due to the antioxidative effects of GB components, particularly polysaccharides (LBPs). Although our study did not directly examine the involvement of specific pathways, previous research has shown that LBPs can modulate the PI3K-Akt-mTOR and p38-MAPK pathways [82], which are crucial in controlling oxidative stress and inflammation [81]. Similarly, the inhibition of the p38-MAPK pathway has been linked to reduced inflammatory responses and oxidative stress, further supporting the observed downregulation of GPX [82].

Overall, our study’s findings align with previous findings in rabbit bucks by Brecchia et al. [34], who demonstrated that GB supplementation significantly enhanced semen quality by increasing sperm concentration, motility, and vitality while reducing the percentage of abnormal spermatozoa [34]. These improvements are consistent with our observations of a reduced inflammatory state and a more favorable sperm environment in the epididymis. The enhanced semen quality reported by that study was attributed to the beneficial effects of GBs on the functional activity of the reproductive tract, particularly in the epididymis and testis. Our findings further support the role of GBs in promoting reproductive health by modulating oxidative stress and inflammation, ultimately contributing to improved sperm quality and function.

## 5. Conclusions

This study provides preliminary new insights into the beneficial effects of Goji berry (GB) supplementation on male rabbit reproductive health, particularly in the epididymis. The significant downregulation of TLR4 expression suggests an anti-inflammatory effect, potentially enhancing the local immune environment and reducing inflammatory responses in this organ, which is essential for sperm maturation and immune defense. Additionally, the lower expression of GPX in the GB-supplemented group indicates an improved antioxidant balance, likely reducing the need for endogenous antioxidant enzymes. These findings suggest that GB supplementation modulates the expression of immune-related and antioxidant genes in the epididymis, promoting a more favorable environment for sperm maturation and storage.

Importantly, this work represents a pilot study and exploratory investigation, laying the groundwork for future research aimed at elucidating the underlying molecular mechanisms and determining the effects of GB supplementation on male fertility. The rabbit model proves to be a valuable system for studying dietary interventions and their impact on male reproductive physiology, offering translational relevance to other species, including humans. Future studies with larger sample sizes are warranted to validate these preliminary findings and to further explore the potential of GB as a natural strategy to enhance male fertility by mitigating oxidative stress and inflammation.

## Figures and Tables

**Table 1:** Formulation of control and 1% of Goji berry-supplemented diets. Ingredients are expressed as a percentage (%).

Ingredients	Diets	
	Control Group	Goji Group
Wheat bran	30	29.5
Dehydrated alfalfa meal	42	41.5
Barley	9.6	9.6
Sunflower meal	4.6	4.6
Rice bran	4	4
Soybean meal	4	4
Calcium carbonate	2	2
Cane molasses	2	2
Dicalcium phosphate	0.7	0.7
Vitamin–mineral premix ^1^	0.4	0.4
Soybean oil	0.4	0.4
Salt	0.3	0.3
Goji berries	-	1

^1^ Per kg diet: vitamin A 11.000 IU; vitamin D3 2000 IU; vitamin B1 2.5 mg; vitamin B2 4 mg; vitamin B6 1.25 mg; vitamin B12 0.01 mg; alpha-tocopherol acetate 50 mg; biotin 0.06 mg; vitamin K 2.5 mg; niacin 15 mg; folic acid 0.30 mg; D-pantothenic acid 10 mg; choline 600 mg; Mn 60 mg; Fe 50 mg; Zn 15 mg; I 0.5 mg; Co 0.5 mg.

**Table 2:** Chemical composition of control and 1% Goji berry-supplemented diets. Analytical data are expressed as a percentage (%).

	Diet	
Analytical Component	Control	Goji
Crude protein	15.74	15.64
Ether extract	2.25	2.23
Ash	9.28	9.36
Starch	16.86	17.07
NDF	38.05	38.55
ADF	19.54	19.6
ADL	4.01	4.31
Digestible energy ^1^	2464	2463

^1^ As Kcal/kg estimated by Maertens et al. [37].

**Table 3:** Oligonucleotide primer sequences for SYBR green quantitative real-time PCR amplification.

Gene	Protein	Gene Bank GI Number	Primer Sequence
ACTB	Actin beta	100009272	F: ACATGGAGAAGATCTGGCAC
			R: GCGTGTTGAACGTCTCGAAC
TLR4	Toll-like receptor–4	100009497	F: TGCATGTCTCAGAACTGCAC
			R: GGATAGGGTTTCCTGTCAATATC
IL-1β	Interleukin-1β	100008990	F: ACAACAAGAGCTTCC
			R: GTGTTGCAGAGGACG
IL-10	Interleukin-10	100008701	F: GCTATGTTGCCTGGTCTTCC
			R: GCTGTTCAGCTGATCCTTCG
TNFα	Tumor necrosis factor	100009088	F: GTGGCCCAGATGGTCACC
			R: CTACTACGTGGGCTAGAGGCTT
SOD1	Superoxide dismutase 1	100009313	F: CACTCCGAGCAGAAGGGAAC
			R: CGTGCCTCTCTTCATCCTTC
CAT	Catalase	100340891	F: GCTGAGATTGAACAGTTGGC
			R: GGTGAGTATCGGGATAGGAG
GPX	Glutathione peroxidase 1	100009258	F: CAGTTTGGGCATCAGGAGAAC
			R: GCATGAAGTTGGGCTCGAAC

## Data Availability

The original contributions presented in this study are included in the article.

## Abbreviations

The following abbreviations are used in this manuscript:

ACTB	Actin beta
TLR4	Toll-like receptor 4
IL-1β	Interleukin-1β
IL-10	Interleukin-10
TNFα	Tumor necrosis factor alpha
SOD1	Superoxide dismutase 1
CAT	Catalase
GPX	Glutathione peroxidase
